# Pilot study of PET imaging of ^124^I-iodoazomycin galactopyranoside (IAZGP), a putative hypoxia imaging agent, in patients with colorectal cancer and head and neck cancer

**DOI:** 10.1186/2191-219X-3-42

**Published:** 2013-06-03

**Authors:** Joseph A O’Donoghue, José G Guillem, Heiko Schöder, Nancy Y Lee, Chaitanya R Divgi, Jeannine A Ruby, John L Humm, Steven A Lee-Kong, Eva M Burnazi, Shangde Cai, Sean D Carlin, Tobias Leibold, Pat B Zanzonico, C Clifton Ling

**Affiliations:** 1 Department of Medical Physics, Memorial Sloan-Kettering Cancer Center, New York, NY 10065, USA; 2 Department of Surgery, Memorial Sloan-Kettering Cancer Center, New York, NY 10065, USA; 3 Department of Radiology, Memorial Sloan-Kettering Cancer Center, New York, NY 10065, USA; 4 Department of Radiation Oncology, Memorial Sloan-Kettering Cancer Center, New York, NY 10065, USA; 5 Department of Radiology, Columbia University Medical Center, New York, NY 10027, USA; 6 Department of Surgery, Columbia University Medical Center, New York, NY 10027, USA; 7 Department of General and Visceral Surgery, Robert-Bosch Hospital, Stuttgart 70376, Germany

**Keywords:** Hypoxia, Iodine-124, IAZGP, PET imaging, 2-Nitroimidazole, Radiation dosimetry, Head and neck cancer, Colorectal cancer

## Abstract

**Background:**

Hypoxia within solid tumors confers radiation resistance and a poorer prognosis. ^124^I-iodoazomycin galactopyranoside (^124^I-IAZGP) has shown promise as a hypoxia radiotracer in animal models. We performed a clinical study to evaluate the safety, biodistribution, and imaging characteristics of ^124^I-IAZGP in patients with advanced colorectal cancer and head and neck cancer using serial positron emission tomography (PET) imaging.

**Methods:**

Ten patients underwent serial whole-torso (head/neck to pelvis) PET imaging together with multiple whole-body counts and blood sampling. These data were used to generate absorbed dose estimates to normal tissues for ^124^I-IAZGP. Tumors were scored as either positive or negative for ^124^I-IAZGP uptake.

**Results:**

There were no clinical toxicities or adverse effects associated with ^124^I-IAZGP administration. Clearance from the whole body and blood was rapid, primarily via the urinary tract, with no focal uptake in any parenchymal organ. The tissues receiving the highest absorbed doses were the mucosal walls of the urinary bladder and the intestinal tract, in particular the lower large intestine. All ^124^I-IAZGP PET scans were interpreted as negative for tumor uptake.

**Conclusions:**

It is safe to administer ^124^I-IAZGP to human subjects. However, there was insufficient tumor uptake to support a clinical role for ^124^I-IAZGP PET in colorectal cancer and head and neck cancer patients.

**Trial registration:**

ClinicalTrials.gov NCT00588276

## Background

There is substantial evidence that tumor hypoxia is an important indicator of clinical prognosis for human cancer. The hypoxic tumor phenotype is complex; its various aspects include reduced sensitivity to low LET radiation, differential patterns of gene expression, enhanced angiogenesis, and an increased propensity to metastatic dissemination [1-8]. Although a full understanding of all the implications of hypoxia has yet to be achieved, relatively simple polarographic probe measurements in accessible tumors typically show a difference in survival between patients with hypoxic tumors and those with non-hypoxic tumors [9-11]. It follows that a non-invasive imaging method, even if it could provide only the same information as polarographic probes, could potentially help predict tumor behavior and enable individualized courses of treatment to be designed on a rational basis.

Positron emission tomography (PET) using hypoxia-selective radiotracers has been proposed as a non-invasive method of visualizing hypoxia within solid tumors. However, the optimal radiotracer has yet to be identified. Typical candidate radiotracers for hypoxia imaging are radiolabeled small molecules that freely diffuse into (and out of) cells but are differentially metabolized in regions of low pO_2_. In hypoxic cells, the molecules are irreversibly reduced and their radiolabeled metabolites bind to macromolecules, resulting in intracellular trapping. Conversely, in non-hypoxic cells, the molecules remain operationally intact and diffuse back out again. Achieving a hypoxia imaging differential requires that the radiolabeled tracer accumulates in sufficient quantity in hypoxic regions while clearing relatively quickly from normoxic regions.

However, hypoxia in tumors is, at least partly, a consequence of a dysregulated and chaotic vascular network leading to an imbalance between oxygen supply and demand. It is likely that this dysfunctional vasculature will also impose some restriction on the delivery of molecular tracers for hypoxia imaging. In contrast, the supply of such tracers to better-vascularized non-hypoxic tumor regions is likely to be greater. Accordingly, the intratumoral distribution of a hypoxia tracer would be expected to undergo a kinetic evolution from an initial state, where it is dominated by vascular supply (higher tracer concentrations in non-hypoxic regions), toward a final state, where it is dominated by the accumulation of trapped metabolites (higher concentrations in hypoxic regions). The hypoxia selectivity of such tracers would thus be expected to increase with time.

There are, however, practical limits to the length of time that is available between tracer administration and imaging. The most commonly used 2-nitroimidazole-based hypoxia radiotracers (^18^F-fluoromisonidazole [12,13] (^18^F-FMISO), ^18^F-fluoro-azomycin-arabinoside [14,15] (^18^F-FAZA), ^18^F-2-(2-nitro-1H-imidazol-1-yl)-*N*-(2,2,3,3,3-pentafluoropropyl)-acetamide [16,17] (^18^F-EF5), ^18^F-fluoro-erythronitroimidazole [18,19] (^18^F-FETNIM)) are all labeled with the positron-emitting radionuclide fluorine-18 (^18^F), and its relatively short (110 min) half-life limits the time for PET image acquisition to a maximum of approximately 3 h post-injection. This may be insufficient to produce a static image that reflects only tumor hypoxia. In principle, the longer (12.6 h) half-life of ^64^Cu allows later imaging times for the candidate hypoxia radiotracer ^64^copper-diacetyl-bis(N4)-methylthiosemicarbazone (^64^Cu-ATSM). However, current clinical imaging studies of ^64^Cu-ATSM typically involve early (<1 h post-injection) imaging [20,21], partly due to historical experience with positron-emitting isotopes of Cu with shorter half-lives (e.g., ^60^Cu = 24 min, ^62^Cu = 10 min) [22,23].

As an alternative to static imaging, kinetic analysis of dynamically acquired PET data has been proposed for assessment of tumor hypoxia [24,25]. This enables generation of parametric images of uptake rate constants or time-activity curve slopes which may, due to the deconvolution of ‘supply’ (i.e., vascular functionality) and ‘demand’ (i.e., hypoxic bioreduction), provide a more accurate representation of tumor hypoxia. This approach, however, has problems of its own related to the long and complex imaging protocols required, image noise, and the accurate determination of arterial input function.

Based on the foregoing considerations, we decided to investigate the use of a long-lived radiolabeled 2-nitroimidazole compound, ^124^I-iodoazomycin galactopyranoside (^124^I-IAZGP) to enable late static imaging of tumor hypoxia. The positron-emitting radionuclide ^124^I has a half-life of 4.2 days enabling, in principle, imaging at times in excess of 24 h post-injection. Additionally, a series of *in vitro* studies with tumor cells in culture and *in vivo* studies in murine xenograft models provided support for the hypothesis that ^124^I-IAZGP might be a superior radiotracer for non-invasive imaging of tumor hypoxia using late PET imaging [26-28]. In order to study ^124^I-IAZGP in human subjects, an investigational new drug application (IND 72,013) was submitted to the US Food and Drug Administration (FDA). Subsequently, we performed a clinical study to evaluate the safety, biodistribution, and imaging characteristics of ^124^I-IAZGP in a group of patients with advanced colorectal cancer and head and neck cancer using serial PET imaging. We now present the results of this clinical study.

## Methods

### Clinical study with ^124^I-IAZGP

Under the auspices of a protocol approved by the Institutional Review Board and an Investigational New Drug application approved by the FDA, ten patients (eight males, two females) with either colorectal or head and neck cancer were intravenously administered 1 mg of IAZGP labeled with ^124^I after having provided informed consent (trial registration ID NCT00588276). Patients underwent serial whole-torso (head/neck to pelvis) PET imaging together with multiple whole-body counts and blood draws. Potential thyroid uptake of ^124^I was blocked by oral administration of potassium iodide (ten drops of SSKI) initiated 2 days before and continued for 2 days after administration of the radiotracer.

### Radiotracer

A single lot of cold precursor stable iodoazomycin galactopyranoside (IAZGP also known as 1-(6-deoxy-6-iodo-β-D-galactopyranosyl)-2-nitroimidazole), synthesized in accordance with cGMP principles by Dr. R.F. Schneider as described previously [26], was used for all clinical studies. IAZGP was radiolabeled with ^124^I produced on an in-house cyclotron (TR19/9, EBCO Technologies, Inc., Vancouver, Canada) by exchange labeling and purified by AgCl-treated celite/anion exchange chromatography as described previously [26]. The radiolabeled drug product was formulated as 1.0 mg IAZGP in sterile physiological saline (pH 6 to 8). Specific activity was nominally 148 MBq/mg (4 mCi/mg) with a radiochemical purity >95%.

### PET imaging

The initial plan was to perform serial whole-torso ^124^I-IAZGP PET scans in a series of ten patients with each patient administered 1 mg of IAZGP labeled with 148 MBq (4 mCi) of ^124^I. Following discussions with the FDA, the first three patients were administered a reduced activity (nominally 111 MBq:3 mCi) of ^124^I, and a provisional analysis was performed before further patient accrual. The provisional analysis led us to modify the imaging scheme for the remaining patients in the study. Specifically, the first three patients were imaged on three to four occasions at nominal times of 3, 18 to 24, and 48 h following injection of 111 MBq of ^124^I-IAZGP (imaging scheme A). The seven remaining patients were imaged on three occasions at nominal times of 3, 6 to 8, and 24 h following injection of 148 MBq of ^124^I-IAZGP (imaging scheme B). For the first three patients, at least one scan was a PET/computer tomography (CT) scan (GE Discovery LS, Waukesha, WI, USA) with the remaining scans as PET only (GE Advance). For the subsequent seven patients, all scans were PET/CT scans (GE Discovery LS, *n* = 1; GE Discovery ST, *n* = 1; GE Discovery STE, *n* = 5). Torso (head/neck to pelvis) PET scans, typically comprising 6 to 7 bed positions, were acquired in two-dimensional (2D) mode with attenuation, scatter, and other standard corrections applied to yield quantitative (Bq/ml) PET images. ^124^I-IAZGP PET scans were interpreted by a board-certified nuclear medicine physician with lesions, identified from contemporaneous ^18^F-fluorodeoxyglucose (FDG) PET scans, scored as either positive or negative for ^124^I-IAZGP uptake.

### Absorbed dose estimates for ^124^I-IAZGP

Absorbed doses to normal tissues for ^124^I-IAZGP were estimated using measured whole-body and blood clearance data, together with uptake data derived from region-of-interest (ROI) analysis of the serial PET scans. Dose estimates were generated using organ-level internal dose assessment/exponential modeling (OLINDA/EXM) [29] for either standard ‘Adult Male’ or ‘Adult Female’ model as appropriate. The input data consisted of cumulative activity per unit administered activity (also known as residence time) for ^124^I-IAZGP for a set of source organs, as discussed in detail below.

### Whole-body and blood clearance

Whole-body clearance was determined by serial measurements of count rate by a NaI(Tl) scintillation probe. Duplicate anterior and posterior measurements were made at fixed geometry, and background-corrected geometric mean values were used for clearance curve fitting. Probe measurements were made immediately post-administration before first voiding, after each voiding over a period of approximately 3 h post-administration, and subsequently at the times of PET imaging. The median number of whole-body count rate measurements was 7 (range, 5 to 10). Count rates were normalized to the value immediately post-administration (taken as 100%) to yield relative retained activities (%).

A median of 10 (range, 9 to 10) venous blood samples (approximately 5 ml) were drawn at nominal times of 5, 15, 30, and 60 min after administration of ^124^I-IAZGP, and before and after each scan up to the nominal 24-h time point. Aliquots (500 μl) of blood or serum were counted in duplicate in a NaI(Tl) gamma well-type detector (Wallac Wizard 1480 automatic gamma counter, PerkinElmer, Waltham, MA, USA) calibrated for ^124^I, the net count rates converted to activities and the results expressed in terms of percentage of the injected dose per unit volume (%ID/l). It was verified, using multiple patient samples, that whole blood and serum activity concentrations were equivalent.

Bi-exponential functions were fitted to the whole-body and blood clearance data using the software application SAAM II (University of Washington, Seattle, WA, USA).

### Parenchymal organs (liver, kidneys, spleen, lung, and thyroid)

Multiple ROIs were drawn on several transverse CT slices for each of the liver, kidneys, spleen, lung, and thyroid and copy/pasted onto the corresponding PET slices. The average activity concentrations in each ROI (Bq/ml) were recorded, and the overall average values were derived for each organ. The total activity in each organ was estimated by multiplying the activity concentration by standard male or female organ masses (i.e., the masses used in OLINDA/EXM). The density of all organs was assumed to be 1 g/ml, except for the lung which was assumed to be 0.3 g/ml. Total cumulated activities in the organs were estimated by trapezoidal integration of the activity-time curve and expressed as cumulative activity per unit administered activity (h) for input into OLINDA/EXM.

### Gastrointestinal tract

The fraction of activity clearing via the gut was estimated from the PET scans acquired the day after administration of ^124^I-IAZGP. It was assumed that all activity clearing via this route would be present in the gut at this time, based on respective nominal transit times of 4, 13, and 24 h for the small, upper large, and lower large intestines [30]. PET images were reformatted to generate summed coronal and sagittal projections, and ROIs were drawn to encompass the entire visible intestine; ROI counts were then expressed as a fraction of the total counts in the patient. The fraction of administered ^124^I-IAZGP present in the intestine was estimated by multiplying the average of the coronal and sagittal count fractions by the decay-corrected whole-body activity at the time of scanning (derived from the whole-body clearance curve). This was then used as input to the ICRP 30 gastrointestinal (GI) module of OLINDA/EXM to yield cumulative activity per unit administered activity for the small, upper large, and lower large intestines.

### Cardiac contents and red marrow

Estimates of cumulative activity per unit administered activity for cardiac contents and the red marrow were derived from the blood clearance curve. The volume of the cardiac contents was taken as 510 ml for males and 370 ml for females, based on standard values [31]. Red marrow mass was taken as 1,120 g for males and 1,300 g for females, corresponding to the values used in OLINDA/EXM. Given the equivalent activity concentrations between whole blood and serum and the implication of unhindered trans-plasma membrane transport of ^124^I-IAZGP, the conservative assumption was made that the activity concentration in the red marrow was equal to that in the whole blood.

### Urinary bladder

It was assumed that any material not cleared via the GI tract was cleared via the urinary bladder. As this corresponded to the majority of administered ^124^I-IAZGP, the conservative assumption was made that the pharmacokinetics of urinary clearance was identical to the bi-exponential whole-body clearance, and these parameters (fractions and biological half-times) were used in the voiding bladder model of OLINDA/EXM.

## Results and discussion

### Results

#### *PET imaging with ^124^I-IAZGP*

**Figure 1:**
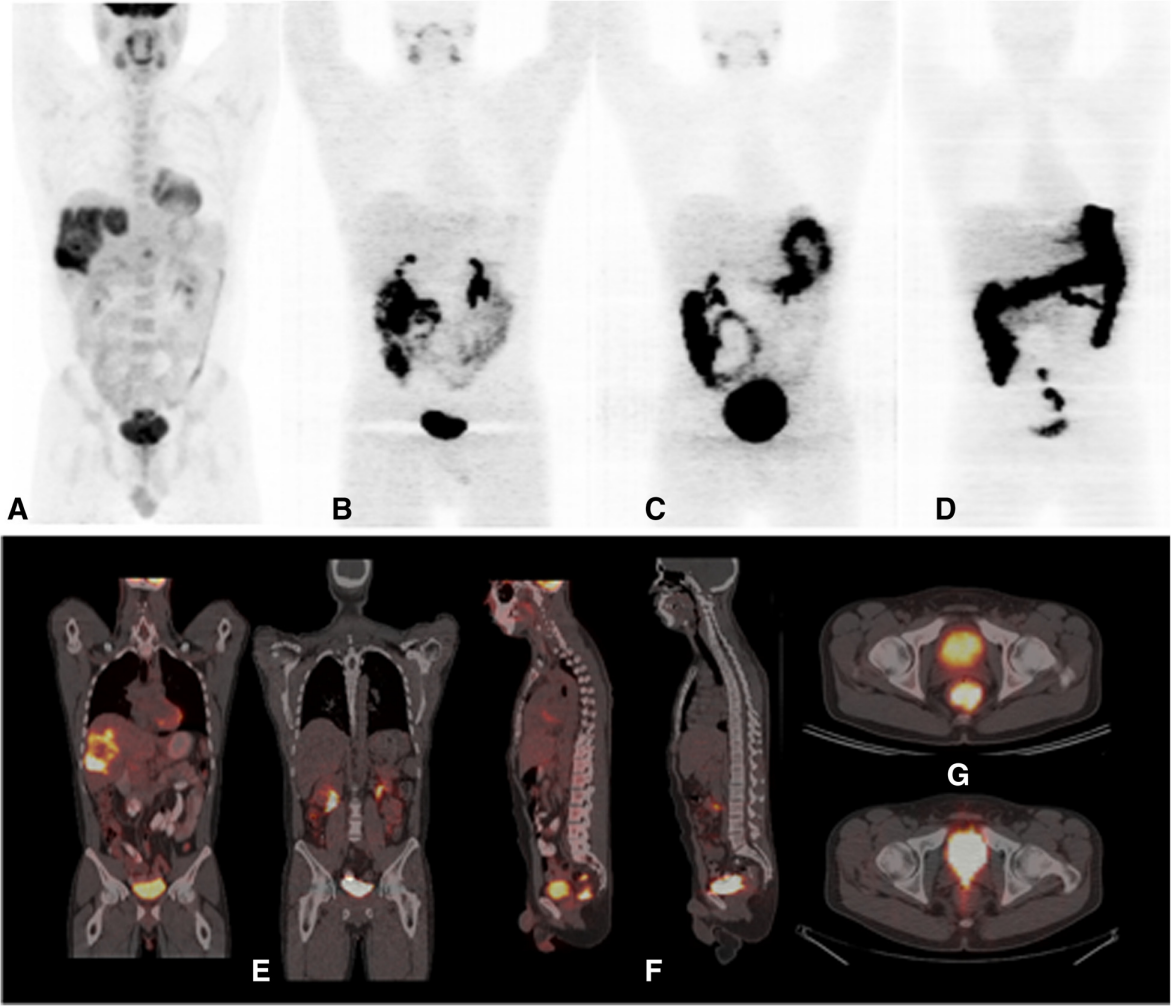
**PET/CT images of **^**18**^**F-FDG and **^**124**^**I-IAZGP in a rectal cancer patient.** (**A** to **D**) Coronal maximum intensity projection PET images of ^18^F-FDG acquired 3 days before ^124^I-IAZGP administration (**A**) and ^124^I-IAZGP acquired at 3 h (**B**), 6 h (**C**), and 25 h (**D**) post-administration in a patient with primary rectal cancer with liver metastases. (**E** to **G**) Cross-sectional fused PET/CT images of the same patient comparing ^18^F-FDG (**E** and **F** left, **G** top) with ^124^I-IAZGP at 3 h (**E** and **F** right, **G** bottom). No evidence of ^124^I-IAZGP uptake is seen in either FDG-avid primary or metastatic lesions. The ^124^I-IAZGP MIP images (**B** to **D**) also illustrate the characteristic pattern of ^124^I-IAZGP clearance, initially via the urinary tract with longer term excretion via the GI tract. Note that image intensities are not directly comparable as window levels were adjusted to maximize feature visibility.

**Figure 2:**
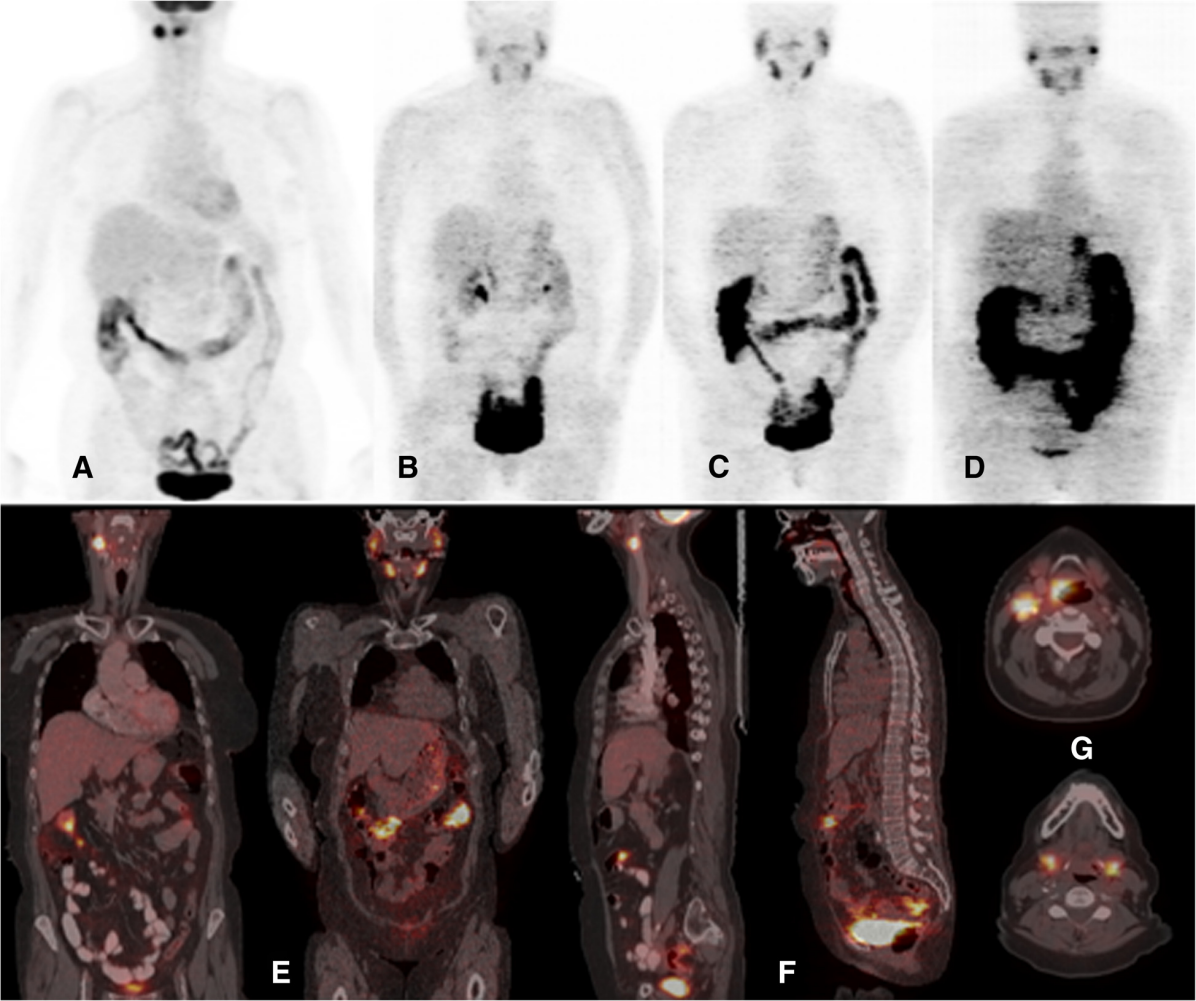
**PET/CT images of **^**18**^**F-FDG and **^**124**^**I-IAZGP in a head and neck cancer patient.** (**A** to **D**) Coronal maximum intensity projection PET images of ^18^F-FDG acquired 3 days before ^124^I-IAZGP administration (**A**) and ^124^I-IAZGP acquired at 3 h (**B**), 6 h (**C**), and 27 h (**D**) post-administration in a patient with head and neck cancer. (**E** to **G**) Cross-sectional fused PET/CT images of the same patient comparing ^18^ F-FDG (**E** and **F** left, **G** top) with ^124^I-IAZGP at 6 h (**E** and **F** right, **G** bottom). No evidence of ^124^I-IAZGP uptake is seen in either the FDG-avid supraglottic larynx cancer or the approximately 2-cm lymph node. The clearance of ^124^I-IAZGP is similar to that seen in Figure 1, with a symmetric distribution of physiologic ^124^I in the salivary glands. Note that image intensities are not directly comparable and that the ^18^F-FDG image was acquired in the radiotherapy treatment position with the patient in a facial mask.

Ten patients completed the study, including five patients with colorectal cancer, four patients with head and neck cancer, and one patient with pseudomyxoma peritonei (Table 1). No adverse effects related to the administration of ^124^I-IAZGP were observed in any patient. ^18^F-FDG PET scans acquired shortly before ^124^I-IAZGP administration indicated FDG-avid disease in all patients. However, all ^124^I-IAZGP PET scans were interpreted as negative, i.e., there was no differential or focal uptake of ^124^I-IAZGP in any disease site at any time. Figure 1 shows an example of a patient with FDG-avid primary rectal cancer with widespread FDG-avid liver metastases, indicating the absence of ^124^I-IAZGP uptake in any sites of disease. Figure 2 shows a patient with head and neck cancer where, again, ^124^I-IAZGP uptake is not observed in FDG-avid disease. Additionally, as illustrated in both figures, there was no focal ^124^I-IAZGP uptake in any parenchymal organ in any patient at any time. Physiologic/excreted ^124^I was seen in the urinary and GI tracts and the salivary glands.

**Table 1:** Patient characteristics

**Patient**	**Tumor site**	**Histologic subtype**	**Differentiation**	**TNM**	**IAZGP (MBq)**	**Imaging scheme**	**IAZGP uptake**	**Neoadjuvant therapy**	**Response (%)**
1	Pseudomyxoma peritonei	Mucinous adenocarcinoma	Moderate	N/A	116	*A*	(-)	None	N/A
2	Tonsil	Squamous	Moderate to poor	cT_1_N_2_M_0_	108	*A*	(-)	Radiation	NED
3	Tonsil	Squamous	Poor	cT_4_N_2_M_0_	59	*A*	(-)	CRT	NED
4	Tongue	Squamous	Poor	cT_1_N_1_M_0_	160	*B*	(-)	CRT	NED
5	Rectum	Mucinous adenocarcinoma	Poor	pT_4_N_2_M_0_	148	*B*	(-)	CRT	95%
6	Rectum	Adenocarcinoma	Moderate	pT_3_N_0_M_1_	141	*B*	(-)	Chemotherapy	50%
7	Rectum	Adenocarcinoma	Moderate	pT_3_N_0_M_0_	150	*B*	(-)	CRT	60%
8	Rectum	Adenocarcinoma	Moderate	cT_4_NxM_1_	159	*B*	(-)	Chemotherapy	Progression
9	Supraglottic larynx	Squamous	Moderate	cT_3_N_2_M_0_	157	*B*	(-)	CRT	NED
10	Sigmoid	Adenocarcinoma	Moderate	cTxN_2_M_1_	159	*B*	(-)	Chemotherapy	Progression

Serial whole-body, blood, and PET data were acquired for patients 1 to 10. Post-imaging clinical management and response are also shown. *CRT* chemoradiotherapy, *NED* no evidence of disease. Imaging schemes: *A* = 3, 18 to 24, and 48 h post ^124^I-IAZGP injection; *B* = 3, 6 to 8, and 24 h post ^124^I-IAZGP injection.

#### *Biokinetics of ^124^I-IAZGP*

Clearance of ^124^I-IAZGP from the whole body and blood was very rapid and was adequately described by bi-exponential clearance curves of the form *A*(*t*) = *A*_1_ exp(-*a*_1_*t*) + *A*_2_ exp(-*a*_2_*t*). The best-fit parameters for the whole body indicated that, on average, two thirds of the activity cleared with an effective half-time of 1 h and the remaining one third with an effective half-time of 11 h (Table 2). For blood, the average effective *T*_1/2_ values for the faster and slower clearance components were 0.24 and 5.2 h, respectively (Table 2). Biological clearance was primarily via the urinary tract with urinary bladder and, to a lesser extent, renal components visible at early imaging times. At later times (approximately 24 h post-administration), a significant proportion (up to 50%) of total activity was visible in the intestines. However, by this time, the amount of activity present in the whole body was only a small fraction (<10%) of that administered. The fraction of ^124^I-IAZGP clearing via the intestinal route was estimated to be, on average, 0.03 (range, 0.02 to 0.05) of that administered (Table 2).

**Table 2:** Kinetic clearance parameters for patients

**Patient #**	**1**	**2**	**3**	**4**	**5**	**6**	**7**	**8**	**9**	**10**	**Mean**	**SD**
WB *A*_1_	0.67	0.89	0.86	0.70	0.80	0.50	0.67	0.28	0.71	0.67	0.67	0.18
WB *a*_1_ (h^-1^)	0.29	0.24	0.38	1.11	0.45	2.10	0.53	0.89	0.52	0.98	0.75	0.56
WB *A*_2_	0.33	0.11	0.14	0.30	0.20	0.50	0.33	0.72	0.29	0.33	0.33	0.18
WB *a*_2_ (h^-1^)	0.06	0.02	0.03	0.06	0.05	0.09	0.08	0.08	0.06	0.10	0.06	0.03
Blood *A*_1_ (%/l)	5.9	18.7	5.7	2.2	5.1	7.1	3.5	2.5	3.2	2.6	5.7	4.9
Blood *a*_1_ (h^-1^)	5.1	6.5	5.8	0.8	1.3	5.6	0.7	1.4	0.8	1.0	2.9	2.5
Blood *A*_2_ (%/l)	1.3	2.48	1.2	0.38	0.68	0.88	0.88	1.07	0.74	0.61	1.01	0.58
Blood *a*_2_ (h^-1^)	0.12	0.12	0.13	0.08	0.07	0.45	0.07	0.10	0.09	0.12	0.13	0.11
Gut fraction	0.031	0.030	0.027	0.049	0.037	0.033	0.030	0.050	0.047	0.016	0.03	0.02

Whole-body and blood clearance data were fitted to bi-exponential functions viz *A*(*t*) = *A*_1_ exp(-*a*_1_*t*) + *A*_2_ exp(-*a*_2_*t*), where *t* is time in hour. Gut fraction is the estimated fraction of ^124^I-IAZGP present in the intestine at (nominally) 24 h after administration.

#### *Residence times and absorbed dose estimates for normal tissues*

Table 3 shows the values of cumulated activity per unit administered activity (i.e., residence time) estimated for patients 1 to 10 as described in the ‘Methods’ section. The OLINDA/EXM-derived absorbed dose estimates based on these input data, together with the associated effective dose [32] values, are shown in Table 4. This indicates that the tissues receiving the highest absorbed doses were the mucosal walls of the urinary bladder and the intestinal tract, in particular the lower large intestine. Average estimated absorbed doses to the bladder wall (0.27 to 0.69 mGy/MBq:1.0 to 2.5 cGy/mCi) were dependent on the assumed frequency of voiding. The average absorbed dose to the LLI wall was 0.35 mGy/MBq (1.3 cGy/mCi). The highest average absorbed dose estimate to the parenchymal organ was 0.056 mGy/MBq (0.21 cGy/mCi) to the kidney followed by 0.044 mGy/MBq (0.16 cGy/mCi) to the liver. The average absorbed dose estimate to the thyroid was 0.022 mGy/MBq (0.082 cGy/mCi). For an administration of the clinically envisaged activity of 148 MBq (4 mCi) of ^124^I-IAZGP, the average absorbed dose to the parenchymal organs was estimated at 0.47 cGy with the kidney receiving an average absorbed dose of 0.83 cGy.

**Table 3:** Cumulated activity per unit administered activity (residence time) in hours for patients 1 to 10

	**Patient number**										**Mean**	**SD**
	**1**	**2**	**3**	**4**	**5**	**6**	**7**	**8**	**9**	**10**		
Sex	M	M	M	M	M	M	M	M	F	F		
Heart contents	0.061	0.117	0.050	0.037	0.071	0.017	0.089	0.063	0.046	0.029	0.058	0.030
Kidneys	0.112	0.161	0.108	0.035	0.035	0.065	0.043	0.048	0.055	0.037	0.070	0.043
Liver	0.24	0.55	0.53	0.19	0.17	0.13	0.24	0.31	0.17	0.14	0.27	0.15
Lungs	0.068	0.148	0.089	0.058	0.052	0.046	0.074	0.106	0.062	0.046	0.075	0.032
Red marrow	0.13	0.26	0.11	0.08	0.16	0.04	0.19	0.14	0.16	0.10	0.14	0.06
Spleen	0.014	0.034	0.039	0.013	0.011	0.011	0.012	0.013	0.011	0.008	0.017	0.011
Thyroid	0.0011	0.0039	0.0012	0.0009	0.001	0.001	0.002	0.001	0.001	0.001	0.0014	0.001
LLI	0.56	0.54	0.49	0.90	0.62	0.60	0.25	0.93	0.86	0.19	0.59	0.25
SI	0.12	0.12	0.10	0.19	0.13	0.13	0.05	0.20	0.18	0.04	0.13	0.05
ULI	0.35	0.34	0.31	0.57	0.39	0.38	0.16	0.58	0.54	0.12	0.37	0.16
UB cont (1 h)	0.49	0.49	0.50	0.54	0.51	0.56	0.51	0.49	0.51	0.54	0.51	0.03
UB cont (2.5 h)	1.28	1.28	1.35	1.56	1.37	1.60	1.39	1.31	1.38	1.53	1.41	0.12
ROB (1 h)	6.35	6.20	4.84	3.49	4.41	4.14	4.16	6.40	4.21	3.14	4.73	1.19
ROB (2.5 h)	5.55	5.41	3.99	2.47	3.55	3.10	3.28	5.58	3.34	2.15	3.84	1.26

*LLI* lower large intestine, *SI* small intestine, *ULI* upper large intestine, UB cont (*t*), urinary bladder contents for a voiding interval of *t*; ROB (*t*), remainder of body for a bladder voiding interval of *t*.

**Table 4:** **Estimated absorbed dose to normal tissues for **^**124**^**I-IAZGP**

	**Patient number**										**Mean**	**SD**
	**1**	**2**	**3**	**4**	**5**	**6**	**7**	**8**	**9**	**10**		
Sex	M	M	M	M	M	M	M	M	F	F		
Adrenals	0.038	0.043	0.034	0.022	0.027	0.024	0.021	0.039	0.034	0.022	0.030	0.008
Brain	0.025	0.025	0.019	0.013	0.017	0.016	0.015	0.026	0.021	0.015	0.019	0.005
Breasts	0.024	0.025	0.020	0.013	0.017	0.015	0.015	0.025	0.021	0.014	0.019	0.005
Gallbladder wall	0.046	0.053	0.045	0.035	0.036	0.033	0.026	0.054	0.047	0.026	0.040	0.011
LLI wall	0.33	0.32	0.29	0.49	0.35	0.34	0.16	0.52	0.52	0.14	0.35	0.13
Small intestine	0.080	0.080	0.068	0.090	0.074	0.071	0.043	0.108	0.110	0.042	0.077	0.023
Stomach wall	0.039	0.041	0.033	0.027	0.029	0.027	0.023	0.043	0.038	0.022	0.032	0.008
ULI wall	0.16	0.16	0.14	0.23	0.17	0.16	0.08	0.25	0.25	0.07	0.17	0.06
Heart wall	0.043	0.057	0.036	0.024	0.036	0.022	0.020	0.045	0.037	0.025	0.035	0.012
Kidneys	0.083	0.112	0.079	0.036	0.037	0.051	0.021	0.053	0.055	0.035	0.056	0.028
Liver	0.044	0.081	0.074	0.034	0.033	0.027	0.021	0.055	0.043	0.030	0.044	0.020
Lungs	0.028	0.042	0.029	0.019	0.021	0.018	0.018	0.035	0.027	0.019	0.025	0.008
Muscle	0.035	0.035	0.029	0.024	0.027	0.025	0.022	0.038	0.033	0.022	0.029	0.006
Pancreas	0.040	0.044	0.035	0.024	0.029	0.026	0.023	0.043	0.036	0.023	0.032	0.008
Red marrow	0.041	0.049	0.034	0.029	0.035	0.026	0.020	0.046	0.042	0.025	0.035	0.010
Osteogenic cells	0.049	0.053	0.039	0.029	0.037	0.030	0.027	0.052	0.048	0.031	0.039	0.010
Skin	0.024	0.025	0.020	0.015	0.018	0.016	0.015	0.026	0.022	0.015	0.019	0.004
Spleen	0.035	0.055	0.053	0.026	0.025	0.025	0.020	0.035	0.032	0.022	0.033	0.012
Testes	0.039	0.039	0.033	0.029	0.031	0.031	0.027	0.042	-	-	0.034	0.005
Thymus	0.030	0.033	0.024	0.016	0.022	0.018	0.018	0.032	0.026	0.018	0.024	0.006
Thyroid	0.025	0.044	0.022	0.016	0.019	0.016	0.018	0.027	0.021	0.014	0.022	0.009
Total body	0.037	0.039	0.031	0.026	0.028	0.026	0.022	0.041	0.036	0.023	0.031	0.007
UB wall (1 h)	0.25	0.25	0.25	0.26	0.25	0.27	0.24	0.26	0.35	0.35	0.27	0.04
UB wall (2.5 h)	0.58	0.58	0.61	0.69	0.61	0.71	0.61	0.61	0.88	0.95	0.68	0.13
Effective dose (mSv/MBq)	0.097	0.10	0.089	0.11	0.094	0.093	0.061	0.13	0.13	0.072	0.098	0.023

All absorbed dose values are expressed in mGy/MBq. Effective dose is expressed in mSv/MBq [32]. Urinary bladder (UB) wall (*t*) denotes absorbed dose to the bladder wall for a voiding interval of *t*.

## Discussion

This study was designed to evaluate the safety, biodistribution, and imaging characteristics of ^124^I-IAZGP in human patients. In particular, the primary imaging function was to assess the time-dependent biodistribution of ^124^I-IAZGP throughout the whole body, rather than its intratumoral distribution, and image acquisition parameters were, necessarily, suboptimal for this latter role. It is also important to note that, like other tumor hypoxia PET tracers, ^124^I-IAZGP is not a tumor imaging agent *per se*, and its potential clinical role would be to characterize tumors that had been pre-identified by other means. Nevertheless, the fact that no differential uptake of ^124^I-IAZGP was observed in any disease site must be considered disappointing. It is unlikely that this finding is due to the absence of tumor hypoxia in the study patients. For example, Figure 1 shows large volume FDG-avid, centrally cold liver metastases suggestive of significant central necrosis and thus likely to contain perinecrotic hypoxic regions.

There is a major discrepancy between the previous pre-clinical studies of ^124^I-IAZGP, which demonstrated its ability to detect hypoxia in tumor cells both *in vitro* and in small animal models [27,28,33,34], and these clinical findings. One factor in this discrepancy may be related to the dilution effect when transitioning from a small animal to a human subject (in terms of both mg/kg and MBq/kg). Our pre-clinical studies used activities of approximately 16.7 MBq in 25-g mice [27] or approximately 18.5 MBq in 200-g rats [28], corresponding to approximately 100 to 700 MBq/kg. In contrast, patients in this study were injected with approximately 148 MBq corresponding to approximately 2 MBq/kg, a factor of 50 to 350 times less than the pre-clinical studies.

We hypothesized that the concentration of radioactivity in tumors might just be too low to be detected with the clinical PET scan parameters used. In order to address this, after the ten-patient imaging study was complete, we included one additional patient who was pre-scheduled for surgical resection of a sigmoid tumor. For this patient, approximately 300 MBq (8 mCi) ^124^I-IAZGP was administered the day before surgery, clinical PET/CT images were acquired at 6 and 21 h post-administration, and following surgery, the entire surgical specimen was imaged on a microPET scanner. In addition, tumor tissue sections cut from the surgical specimen were exposed to a phosphor imaging plate for up to 7 days for digital autoradiography (DAR). However, not only were the clinical PET scans negative as before, but the *ex vivo* evaluation of the surgical specimen also returned negative results. (Additional file 1: Figure S1 shows the DAR results).

The biodistribution of ^124^I-IAZGP was characterized by rapid clearance from the blood and whole body primarily via the urinary and, to a lesser extent, GI tracts, with no significant uptake in the parenchymal tissues apart from the salivary glands, presumably reflecting dissociated iodine. In terms of radiation exposure, 148 MBq (4 mCi) of ^124^I-IAZGP produced absorbed doses to normal parenchymal tissues less than or equal to those produced by typical clinical activities (370 to 444 MBq; 10 to 12 mCi) of ^18^F-FDG. However, the absorbed doses to the large intestinal and urinary bladder walls were significantly greater for ^124^I-IAZGP than ^18^F-FDG. As absorbed doses to the urinary bladder wall depend on the frequency of voiding, study patients were encouraged to hydrate and to void frequently in order to minimize this.

There is no doubt that the use of long-lived positron-emitting nuclides for imaging has intrinsic dosimetric disadvantages compared to short-lived radionuclides, where the balance between image quality and patient dose is usually more favorable. However, the use of a long half-life radionuclide is unavoidable if it takes a long time for sufficient biological contrast to develop; our hypothesis was that PET imaging of tumor hypoxia was such a case. An additional disadvantage associated with ^124^I is its relatively low positron yield (approximately 24%) coupled with the presence of multiple high-energy cascade gamma rays which act to further decrease the ratio of PET image quality to patient radiation dose.

We found that PET imaging of ^124^I-IAZGP in 2D mode was possible up to, at least, 6 to 8 h post-administration and possibly 24 h, although with an enhanced likelihood of low-count reconstruction artifacts. However, imaging at 48 h was generally not feasible with the activities (approximately 148 MBq) and emission times (typically 6 to 8 min per bed position) used in this study. This contrasts to the case where ^124^I is used to label antibodies with prolonged biological retention, for which clinically useful images may be acquired at times of up to 1 week post-administration [35,36]. This deficiency may be alleviated to some extent by the use of 3D reconstruction methods as long as appropriate corrections are included for cascade coincidences.

However, even with foreseeable improvements in image acquisition and reconstruction methods, our data indicate the uptake of ^124^I-IAZGP in tumors is insufficient to suggest any clinical role in the management of patients with colorectal or head and neck cancer.

## Conclusions

The study reported here is the first trial describing the toxicity and biodistribution of ^124^I-IAZGP in human subjects. We found that the administration of ^124^I-IAZGP was safe and that it was rapidly cleared from the body. PET imaging failed to show any differential uptake of ^124^I-IAZGP in any disease site. While we continue to believe that characterization of tumor hypoxia will be important for individualizing patient treatments, ^124^I-IAZGP PET in its current form is unlikely to provide any clinical utility for assessing hypoxia in colorectal cancer and head and neck cancer. Despite these negative results with ^124^I-IAZGP, there is still a strong theoretical rationale for the concept that late PET imaging should provide the most specific indication of tumor hypoxia. It is hoped that new hypoxia agents will emerge, perhaps featuring alternative long-lived positron-emitting radionuclides such as ^64^Cu, ^89^Zr, or ^76^Br, which can provide a convenient way to perform single time-point imaging of tumor hypoxia.

## Competing interests

The authors declare that they have no competing interests.

## Authors’ contributions

JAOD, JGG, HS, NYL, CRD, JLH, PBZ, and CCL participated in the concept, design, and analysis of the study. JAOD, JLH, SDC, and PBZ acquired the data. JGG, NYL, JAR, SALK, and TL were responsible for primary clinical management and patient accrual. EMB and SC participated in radiotracer development, formulation, and quality assurance. JAOD and JAR drafted the manuscript. All authors read and approved the final manuscript.

## Supplementary Material

Additional file 1: Figure S1 Representative frozen sections from patient surgical specimen showing (left) H&E, (center) digital autoradiogram without digital manipulation, i.e., each section is quantitatively comparable, and (right) digital autoradiogram optimized to maximize contrast. The exposure time for the autoradiograms was 4 days. Overall, the absolute amount of ^124^I in the tumor is low (compare signal/background in center) and relatively uniform with no enhancement in tumor uptake compared to normal colon. Click here for file
