# Supplementary figures and images for: Pilot study of PET imaging of 124I-iodoazomycin galactopyranoside (IAZGP), a putative hypoxia imaging agent, in patients with colorectal cancer and head and neck cancer
Source: EJNMMI Res. 2013 Jun 3;3:42. doi: 10.1186/2191-219X-3-42 (PMC3686612; doi:10.1186/2191-219X-3-42)

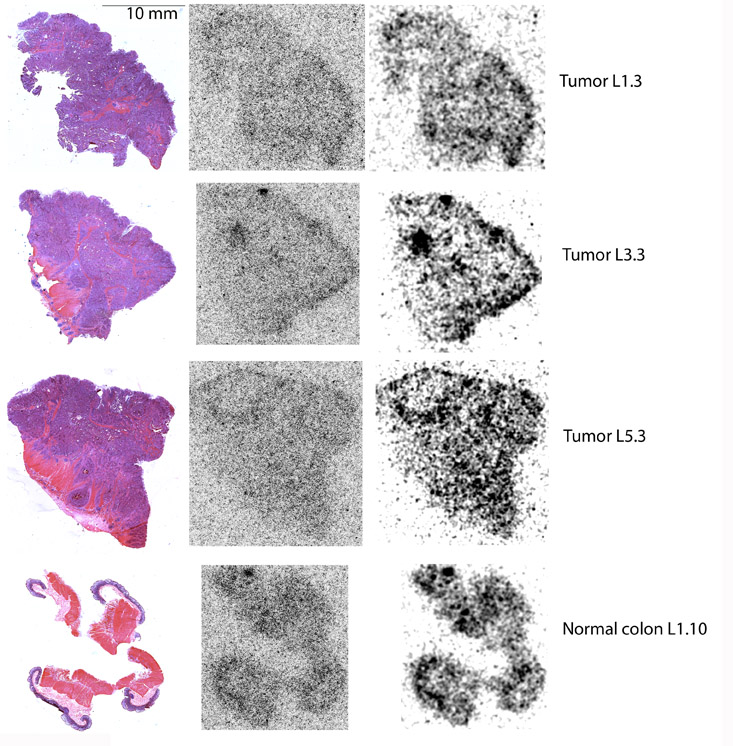

Supplement: Additional file 1: Figure S1 — Representative frozen sections from patient surgical specimen showing (left) H&E, (center) digital autoradiogram without digital manipulation, i.e., each section is quantitatively comparable, and (right) digital autoradiogram optimized to maximize contrast. The exposure time for the autoradiograms was 4 days. Overall, the absolute amount of 124I in the tumor is low (compare signal/background in center) and relatively uniform with no enhancement in tumor uptake compared to normal colon. [file 2191-219X-3-42-S1.jpeg]
